# Quantitative metabolomics analysis of amino acid metabolism in recombinant *Pichia pastoris* under different oxygen availability conditions

**DOI:** 10.1186/1475-2859-11-83

**Published:** 2012-06-15

**Authors:** Marc Carnicer, Angela ten Pierick, Jan van Dam, Joseph J Heijnen, Joan Albiol, Walter van Gulik, Pau Ferrer

**Affiliations:** 1 Department of Chemical Engineering, Universitat Autònoma de Barcelona, Bellaterra, (Cerdanyola del Vallès), 08193, Spain; 2 Department of Biotechnology, Delft University of Technology, Julianalaan 67, Delft, 2628BC, The Netherlands

**Keywords:** *Pichia pastoris*, Metabolite quantification, Recombinant protein production, Hypoxia, Amino acids, Metabolic burden

## Abstract

**Background:**

Environmental and intrinsic stress factors can result in the global alteration of yeast physiology, as evidenced by several transcriptional studies. Hypoxia has been shown to have a beneficial effect on the expression of recombinant proteins in *Pichia pastoris* growing on glucose. Furthermore, transcriptional profiling analyses revealed that oxygen availability was strongly affecting ergosterol biosynthesis, central carbon metabolism and stress responses, in particular the unfolded protein response. To contribute to the better understanding of the effect and interplay of oxygen availability and foreign protein secretion on central metabolism, a first quantitative metabolomic analysis of free amino acids pools in a recombinant *P. pastoris* strain growing under different oxygen availability conditions has been performed.

**Results:**

The values obtained indicate significant variations in the intracellular amino acid pools due to different oxygen availability conditions, showing an overall increase of their size under oxygen limitation. Notably, even while foreign protein productivities were relatively low (about 40–80 μg Fab/g_DCW_·h), recombinant protein production was found to have a limited but significant impact on the intracellular amino acid pools, which were generally decreased in the producing strain compared with the reference strain. However, observed changes in individual amino acids pools were not correlated with their corresponding relative abundance in the recombinant protein sequence, but to the overall cell protein amino acid compositional variations.

**Conclusions:**

Overall, the results obtained, combined with previous transcriptomic and proteomic analyses provide a systematic metabolic fingerprint of the oxygen availability impact on recombinant protein production in *P. pastoris*.

## Background

*Pichia pastoris* has emerged as a workhorse for the production of recombinant proteins [1-4]. Moreover, the development of both synthetic and systems biotechnology tools specific for this cell factory platform [5-13], has opened new opportunities for metabolic engineering, as well as rational design and optimization of media composition and culture conditions.

Recombinant protein overproduction often results in a metabolic burden. Such effect may be reflected on process parameters such maximum growth rate, biomass yield or specific substrate consumption of yeast cells [4,14-17], thus suggesting a potential impact on the cell’s energy metabolism, possibly derived from higher maintenance requirements [18]. Furthermore, production of recombinant proteins may cause cellular stress due to unfolded proteins and unsuitable or inefficient secretion [19], which, in turn, may negatively affect cell growth, even at relatively low expression levels [15,20], that is, at the product yields range where effects derived from increased energy and precursor demands for protein synthesis on cell growth should be theoretically negligible. In this context, limited but significant alterations in the carbon flux distribution over the central metabolism have been recently reported [21-24]. Also, amino acid supplementation of the growth medium has been shown to partially unburden cellular metabolism during recombinant protein production in yeast [23,25-27]. Interestingly, such studies in *P. pastoris* have provided novel evidence that the adaptation of the central metabolism to recombinant protein production can not only be explained by an increased drain of precursors for protein synthesis [23]. Indeed, amino acids are not only important precursors for protein synthesis but also participate in the regulation of major metabolic pathways. Glutamic and Aspartic acid for instance, are components of the aspartate/malate redox shuttle [28] and their concentrations may indirectly impact on the rate of oxidation of glycolytic NADH.

We have previously reported the beneficial impact of hypoxia conditions on recombinant protein production in *P. pastoris*[29]. The physiological bases of this beneficial effect were further investigated in a recent multilevel study including transciptome, proteome and metabolic flux analyses [30]. These studies allowed to gather information on the biological processes involved in the adaptation to hypoxia and their relation with extracellular recombinant protein production in *P. pastoris*. However, the potential effect of oxygen availability and/or recombinant protein production on the intracellular metabolite levels (particularly of the amino acid precursors used for protein synthesis) remains to be elucidated.

To investigate the potential impact of foreign protein expression and secretion on amino acid metabolism, the free intracellular amino acids pools were analyzed in carbon limited chemostat cultivations at a fixed growth rate and different oxygenation conditions, using a recombinant *P. pastoris* strain secreting an antibody Fab fragment (E). The results are compared to the reference (non-producing) strain (C) and further combined with the metabolic fluxes related to protein synthesis and global transcriptome dataset from our previous study performed with the same strains and analogous cultivation conditions [30]. Overall, this study aimed at understanding further the behaviour of the variations in intracellular amino acid levels as a result of the different oxygenation conditions employed, as well as gaining further insight in the potential interactions between energy metabolism and amino acid metabolism and, how such interactions may be perturbed by heterologous protein secretion in *P. pastoris*.

## Results and discussions

### Growth and recombinant protein secretion in recombinant *P. Pastoris*

The burden caused by recombinant protein production in yeast and, in particular, *P. pastoris*, has been recently suggested to impact the central metabolism even at relatively low expression levels, *i.e.* where increased precursor (amino acids) demands for recombinant protein production may be negligible [10,23,24]. Integration of transcriptomic, fluxomic and metabolomic data of recombinant cells under different environmental conditions may help to understand the metabolic adaptations of the cell’s central metabolism to protein production under different environmental conditions, particularly in relation to amino acid metabolism. In this study, intracellular amino acid pools of a recombinant *P. pastoris* strain expressing an antibody Fab fragment under the control of a constitutive pGAP promoter were measured during growth in glucose-limited chemostat cultures under different oxygen availability conditions. The macroscopic growth parameters for both the control and Fab-producing *P. pastoris* strains during growth at three different oxygenation levels are given in Table 1. Coherent with previous studies [30,31], the adaptation from normoxic (fully aerobic) to hypoxic conditions lead to a shift from fully respiratory to respiro-fermentative metabolism, as well as increased secreted recombinant product productivities. Importantly, growth parameters for the normoxic and oxygen-limiting conditions were statistically identical to those previously reported by Baumann and co-workers in a transcriptomic, proteomic and fluxomic profiling study of the same strains growing under analogous conditions [30]. Conversely, the hypoxic condition tested in this study appeared to be less stringent compared with the corresponding cultivation condition previously reported by Baumann and co-workers [30], as indicated by a lower respiratory quotient (RQ), as well as lower specific rates of ethanol and arabinitol production (See Additional file 1 for a vis-à-vis comparison of results between this study and data reported in [30]). This was further supported by the fact that, in our previous studies only 3.5 residence times could be accomplished in the hypoxic condition [29-31], whereas in this study the hypoxic condition could be extended up to a minimum of 5 residence times, which is the period required to reach a true metabolomic steady state [32].

**Table 1:** Summary of macromolecular culture parameters

		**Control Strain**			**Expressing Strain**	
	**Normoxic**	**O_2_-limited**	**Hypoxic**	**Normoxic**	**O_2_-limited**	**Hypoxic**
q_Fab_				40 ± 5	82 ± 2	74 ± 9
q_Glc_	−1.00 ± 0.02	−1.28 ± 0.03	−1.72 ± 0.05	−1.01 ± 0.02	−1.37 ± 0.03	−1.56 ± 0.04
q_O2_	−2.35 ± 0.06	−2.01 ± 0.07	−2.01 ± 0.15	−2.44 ± 0.07	−1.99 ± 0.08	−1.81 ± 0.13
q_CO2_	2.43 ± 0.06	2.55 ± 0.06	3.21 ± 0.14	2.52 ± 0.07	2.68 ± 0.07	2.94 ± 0.12
q_X_	3.57 ± 0.15	3.83 ± 0.18	3.77 ± 0.23	3.55 ± 0.15	3.77 ± 0.18	3.58 ± 0.22
q_EtOH_		0.31 ± 0.02	0.84 ± 0.06		0.41 ± 0.03	0.83 ± 0.06
q_Ara_		0.13 ± 0.01	0.33 ± 0.01		0.19 ± 0.01	0.24 ± 0.02
RQ	1.03 ± 0.04	1.27 ± 0.05	1.60 ± 0.14	1.03 ± 0.04	1.34 ± 0.06	1.63 ± 0.13

q_Gluc_ and q_O2_ are specific utilization rates, and q_X_, q_Ara_, q_EtOH_ and q_CO2_ are specific production rates, where Glc, Ara, EtOH and X stand for glucose, arabinitol, ethanol and biomass, respectively. Conversion rates are given in mmol/(g_DCW_·h), except for q_Fab_, which is given in μgFab/(g_DCW_·h) . DCW, dry cell weight. RQ = q_CO2_ / q_O2_.

### Global analysis of intracellular amino acid pools

**Figure 1:**
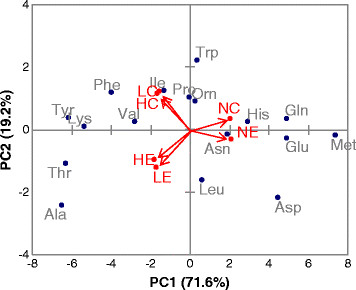
**Principal component analysis (PCA) of amino acid data.** Principal component analysis of the intracellular amino acid pools data in a 2D graph of PC1 and PC2. The biplot shows amino acid data (scores) as labeled dots and treatment effect (loadings) as vectors for the expressing (E) and control (C) strain at different concentrations in the inlet gas (Normoxia (N); Oxygen-limited (L) and Hypoxia (H)). Vectors that are close together are highly correlated in terms of the observed amino acid pool sizes for each treatment, while vectors that are orthogonal are poorly correlated. PC1 correlates well with the change in oxygen conditions, whereas PC2 appears to be correlated with the strain type.

To obtain a global overview of the effects of different oxygen supply conditions and recombinant protein secretion on the measured intracellular free amino acid pool sizes, we subjected the relative changes in metabolite pool sizes to Principal Component Analysis (PCA) (Figure 1; see also Supplementary Material 1 for full data from PCA analysis). PCA projection demonstrated that the maximum variability in the data set clearly differentiated between different oxygenation conditions (precisely, between normoxia and the two oxygen-restricted conditions), with the first component (PC1) covering 71.3% of the data variance. The second principal component (PC2), which explained only 19.2% of the total amino acid pools variance, clearly discriminated between the Fab expressing and the reference strain, indicating a limited impact of the antibody fragment production on the *P. pastoris* amino acid metabolome. Ala, Trp and Asp were the amino acids with the highest contribution (24.0%, 20.6% and 19.7% respectively) to the variance in PC2.

Overall, these data reflect a higher impact of oxygen availability rather than recombinant protein production on the global physiologic response of *P. pastoris*, consistent with previous transcriptomic, proteomic and metabolic flux analyses [30]. To help decipher the potential dependence of the amino acid metabolism on oxygen availability and the burden caused by recombinant protein secretion, a more detailed analysis was performed focusing on each of these two factors separately.

### Recombinant protein production effect

The amino acid pools provide building blocks for protein synthesis. Therefore, an impact of Fab production on these metabolites was *a priori* expected. In fact, previous studies using amino acids for media supplementation or complex extracts have proven to have a positive effect on recombinant protein production [22,27,33].

**Figure 2:**
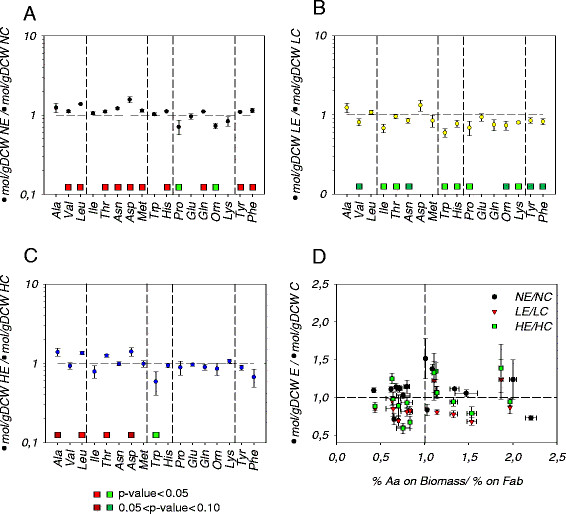
**Amino acid pools comparison of *P. pastoris *control (C) and Fab expressing (E) strains under different oxygen conditions. ** The values are the average and the standard error of calculated ratios. Ratio errors were calculated using error propagation. Horizontal dashed line represents a ratio of 1. In A, B and C vertical dashed line separate metabolites belonging to the same metabolic pathway. The squares over the metabolite names denote the results from the T-Student test. Green and red colors indicate lower and higher ratios respectively. **A**: normoxic condition; **B**: O_2_-limited condition; **C**: hypoxic conditions. **D**: Biomass/Fab amino acid proportion ratios influence on the intracellular amino acid pool changes. N: Normoxic, L: O_2_-limited; H: Hypoxic.

In order to analyze more specifically the potential effects of recombinant protein production on the *P. pastoris* amino acid metabolite levels, a comparison was made of the free amino acid pools of the reference and the Fab producing strains grown at each oxygenation condition. The metabolites levels measured in the control strain grown at each oxygenation condition were taken as reference values to be compared with the metabolites of the Fab-expressing strain grown at the corresponding condition, thereby obtaining concentration ratios for each metabolite of the Fab-expressing relative to the control strain at each culture condition (Figure 2). In addition, two-tailed *T*-test statistical analyses allowed to identify those metabolite ratios that were significantly higher or lower than 1 (that is, those metabolite pool sizes that varied significantly between both strains).

The impact of recombinant protein production on amino acid pool sizes under normoxic conditions is shown in Figure 2A. Overall, 10 out of 17 amino acid pools increased their intracellular levels significantly, while only 2 of them had an opposite behavior. In particular, most of the free amino acids from the Ala, Asp and Phe families displayed significantly increased levels in the Fab-expressing strain. Nevertheless, the Glu family showed different trends depending on the amino acid, being Orn and Pro significantly decreased, while Gln increased. Increased amino acid pools in the Fab-expressing strain were not accompanied by any significant flux redistribution though the central metabolism nor proteome compositional change, when compared to the control strain growing under the same oxygenation condition [30,31].

Conversely, generally decreased amino acid pool ratios (11 out of 17) were found in the Fab-expressing strains under oxygen-limiting conditions, compared to the reference strain (Figure 2B), that is, an opposite behavior to that observed under normoxic conditions. We observed the same trend when comparing the Fab-expressing strain growing under hypoxic condition; however, the data variance was higher and, therefore, the observed changes were not statistically significant. *A priori*, this observation might be associated to the increased specific heterologous protein productivity found under oxygen limitation, as the Fab producing strain might require higher metabolic fluxes of amino acids for protein synthesis, thereby causing a drain of precursor pools. Nevertheless, this hypothesis is highly unlikely, since Fab production levels were relatively low in relation to the total cell protein [31]. Besides, this trend was not uniform within each of the amino acid families. For instance, in the Ala family, Ala and Leu pools increased while Val decreased in the Fab-expressing strain. Also, in the Asp amino acid family only Asp levels varied significantly. Moreover, no correlation was found between the relative abundance of each amino acid in the Fab antibody fragment and the observed changes in the corresponding free amino acid pools (Figure 2D). Nevertheless the specific Fab production correlated inversely (correlation value of −0.93) with the average of the metabolite ratios between Fab-expressing and reference strains at each culture condition. This may reflect a global re-adjustment of the free amino acid pools to compensate for the recombinant protein overproduction. Although such readjustment could not be the result from a direct drain of building blocks at higher Fab synthesis levels, other phenomena related with recombinant protein production might provide some explanations. For instance, recent studies on recombinant protein secretion using ^34^ S labeling strategies with the same strain used in this study have revealed that about 58% of the Fab protein produced intracellularly is actually degraded within the cell, and only 35% is secreted [34]. In addition to protein degradation, increased energetic demands related with the cost of the folding, refolding and secretion processes of the Fab product could result in an overall readjustment of amino acid metabolism. In fact, when the Fab producing strain was cultivated at lower temperatures (20°C), the unfolded protein stress response was reduced, leading to a reduced metabolic burden and higher specific productivities compared with cells grown at 25°C and 30°C, in which an increase in energy demand was evidenced by an up-regulation of the TCA cycle, slightly higher in Fab-secreting strains [10]. Besides, increased maintenance requirements associated with heterologous protein production (*e.g.* unfolded protein stress response) may cause additional energy demands. Interestingly, recent amino acid supplementation studies using a recombinant *P. pastoris* strain revealed that such supplementation partially relieved the metabolic burden from recombinant protein production. Furthermore, de novo amino acid synthesis in cells growing on different combinations of amino acids supplementations was inversely correlated with the corresponding energetic cost for most amino acids [23].

**Figure 3:**
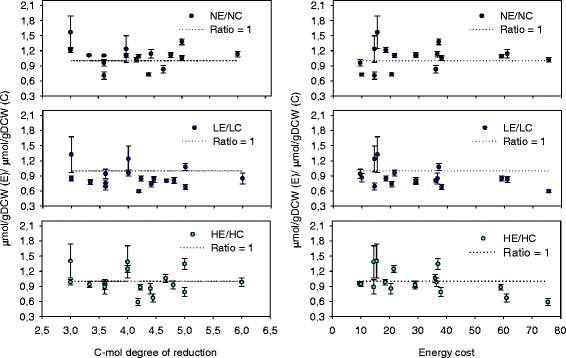
**Amino acid C-mol degree of reduction and energy costs influence on the expressing/control amino acid pool ratios.** The values are the average and the standard error of calculated ratios. Horizontal dashed line represents a ratio of 1. Ratio errors were calculated using error propagation. The energy cost for an amino acid is defined as the number of high energy phosphate bounds (~PO_4_) that are required for its synthesis. The amino acids energy costs were taken from *S. cereviciae* published data [35]. The C-mol degree of reduction of each amino acid was calculated from each molecular formula.

The potential dependence between fold changes observed amongst amino acid pools when comparing the Fab-expressing to the reference strain and their corresponding energy and redox costs were analyzed (Figure 3). Interestingly, lower fold changes in amino acid pools with the highest energy cost (that is, aromatic amino acids family) was found under oxygen reduced conditions (that is, under higher Fab productivities), but not under normoxia. This might point at the hypothesis that the cell adjusts its overall amino acid metabolism to minimize the energetic burden caused by Fab production. Nevertheless, no trend was observed regarding the C-mol degree of reduction of each amino acid (Figure 3).

### Oxygen availability effect

Previous transcriptomic studies revealed amino acid metabolism as one of the major cellular processes regulated by oxygen availability [30]. Interestingly, the number of genes in the gene ontology group of amino acid metabolism that were down regulated under hypoxic conditions was substantially higher in the Fab-producing strain.

**Figure 4:**
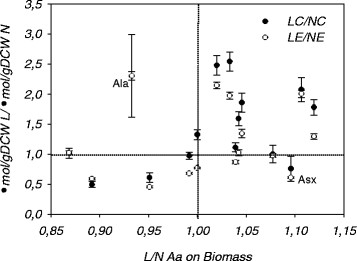
**Impact of the differential biomass demand over the intracellular amino acid pools under different oxygen conditions.** The values are the average and the standard error of calculated ratios. Horizontal and vertical dashed lines divided the graph in four spaces to facilitate the interpretation. The biomass demands under normoxia and oxygen-limited conditions were taken from [31]. Asx = Asp + Asn.

To investigate the potential correlation between observed changes in free amino acid pools and proteome amino acid composition upon oxygen availability changes, we related the observed fold changes for each free amino acid pool with the change in the relative abundance of the corresponding amino acid in the cell’s proteome amino acid composition (Figure 4). The effect of oxygen availability on the cell’s amino acid metabolism could be already inferred from previous measurements of the cell’s protein amino acid composition [31]. For instance, the relative abundance of amino acids derived from cytosolic oxalocetate (OAA), pyruvate (Pyr), phosphoenolpyruvate and 3-phospho glycerate (3PG) were increased and those derived from mitochondrial α-ketoglutarate (αKG) were reduced under hypoxic conditions. Remarkably, such changes in amino acid pools synthesized from glycolytic and tricarboxylic acid (TCA) cycle precursors appeared to be directly correlated with increased glycolytic and decreased oxidative TCA cycle fluxes under reduced oxygen levels, respectively [30,31].

Overall, changes in free amino acid pools seemed to correlate directly with changes in the relative abundance in the cell proteome for most amino acids, suggesting that biosynthetic fluxes to cell protein had a direct impact on the precursor amino acids pools sizes. A clear exception to this observation was Ala and, to a much lesser extent, Asx (Asp + Asn). Unfortunately, the available metabolomic methodology [32] did not allow for accurate quantification of the metabolite precursors of these amino acids (that is, Pyr and OAA), hampering the interpretation of the observed changes in Ala and Asx pool sizes.

**Figure 5:**
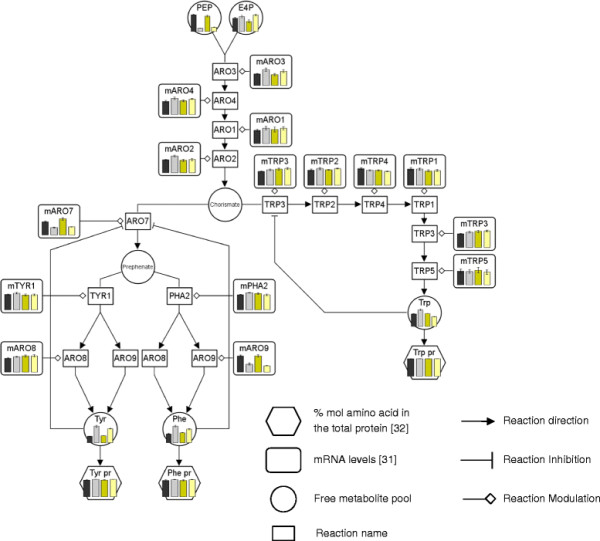
**Aromatic amino acid biosynthetic pathway behaviour under different oxygen conditions.** The metabolite level bars in the graphs are the average and the standard error from at least 4 measurements. The control and the Fab-expressing strain are represented by black-grey and yellow bars respectively. Oxygenation conditions from normoxic to oxygen-limited conditions are represented as light to dark color scale. The mRNA data are indicated as m plus the specific reaction name. Each graph has its own scale.

**Figure 6:**
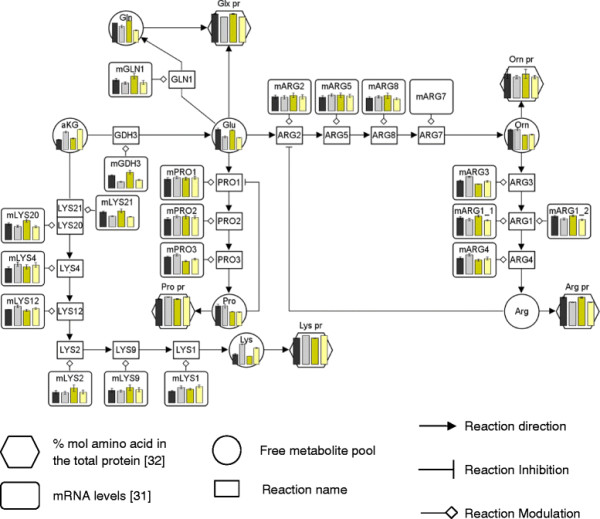
**Glutamate family biosynthetic pathway behaviour under different oxygen conditions.** The metabolite levels bars in the graphs are the average and the standard error from at least 4 measurements. The control and the Fab-expressing strain are represented by black-grey and yellow bars, respectively. Oxygenation conditions from normoxic to oxygen-limited conditions are represented as light to dark color scale. The mRNA data are indicated as m plus the specific reaction name. Each graph has its own scale.

To obtain an overview of the potential correlations between transcriptional changes in amino acid biosynthetic genes and intracellular amino acid levels (free pools as well as proteome amino acids) upon a change in oxygen availability, transcriptomic, metabolomic and cell protein compositional data were integrated into biosynthetic pathway maps (Figures 5 and 6; Additional file 1). The specific amino acid composition of the whole protein extracts at different oxygen conditions was included in order to show the biosynthetic demand (*i.e.* carbon flux) for each specific amino acid synthesis pathway. Since hypoxic condition of this study was not equivalent to that used in previous transcriptomic studies, only normoxic and oxygen limiting conditions were compared for both E and C strains.

A direct comparison of the relative changes observed in the free amino acid levels between different oxygen availability conditions and the corresponding changes observed in the whole cell’s amino acid component (that is, free intracellular amino acid pools plus cell protein amino acids) indicated that the latter were less pronounced.

The integrated data seem to reflect the oxygen-dependent transcriptional regulation of amino acid biosynthesis pathways. In particular, transcriptional levels of several key regulatory enzymes in the biosynthetic pathways correlated inversely with the carbon flow through them, as well as with their corresponding end-metabolite levels, probably denoting the negative feedback control of the pathway. For instance, the levels of free tyrosine and phenylalanine were substantially increased under oxygen-limiting conditions, concomitantly with a reduction of the mRNA levels of *ARO7*, while an opposite pattern was observed for the free tryptophan pools and *TRP3* transcriptional levels (Figure 5). This pattern was also observed in other amino acid biosynthetic pathways such as methionine (see Additional file 2).

Similarly, in the glutamate amino acid family (Figure 6), the carbon flow to lysine was increased under oxygen limiting conditions, concomitantly with a reduction in transcript levels of several genes of its pathway, particularly *LYS20*, the first reaction of the pathway. In addition, the carbon flux to the Glu and Gln biosynthetic pathway was significantly reduced at lower oxygen availability. This pattern correlated directly with the reduced transcriptional levels of *GDH3* and *GLN1*.

Conversely, the reduced Ala and Asp biosynthetic fluxes to cell protein under limited oxygen levels seemed to be directly correlated to *ATL1* and *ATT1/ATT2* transcriptional levels, respectively (Additional files 2 and 3). Also, no significant variation on the His biosynthetic flux was observed despite the significant transcriptional and His pool sizes changes measured upon oxygen limitation.

## Conclusions

Previous transcriptomic studies [30] pointed at the impact of oxygen availability and recombinant protein production on amino acid metabolism. The analysis at the metabolomic level further confirms such impact. In particular, our data point at a major impact of oxygen availability rather than recombinant protein production on the free amino acid pools, coherent with previous transcriptional analyses. Notably, changes in free amino acid pools observed at different oxygenation conditions generally correlated directly with the changes in relative abundances in the corresponding amino acids of the cell’s proteome, with alanine being the major exception. In contrast, the impact of recombinant protein production on the free amino acids pools depended on the oxygenation state. Importantly, the observed changes did not correlate with the difference in amino acid composition of the recombinant product and the cell’s proteome, but rather to the energetic costs (specifically, for those amino acids with highest energy costs), thereby suggesting a possible dependence between mitochondrial metabolism and amino acid anabolism as a potential target to modulate the metabolic burden caused by recombinant protein production.

Systematic integration of metabolomic and transcriptional data into genomic-scale metabolic models should allow gaining further understanding of the behaviour of central and amino acid metabolism, as well as identifying metabolic bottlenecks limiting enhanced recombinant protein production.

## Methods

### Strain and cultivation conditions

Analytical grade reagents were supplied by Sigma-Aldrich. HPLC-grade methanol and ethanol were supplied by J.T. Baker.

In this study, the *P. pastoris* strain X-33 pGAPZαA Fab3H6 [36], secreting the light and heavy chains of a human monoclonal antibody Fab fragment under the constitutive GAP promoter and the *S. cerevisiae* alpha-mating factor leader, was used as expressing strain. A strain with an integrated empty-vector was used as reference strain. The experimental set up was as described in [32]. Briefly, glucose-limited chemostat cultures at a dilution rate of 0.1 h^-1^ at different oxygenation conditions were carried out by changing the oxygen content of the inlet gas. Initially, the oxygen concentration in the inlet gas stream corresponded to normal air (20.95% v/v) leading to a totally normoxic condition (*i.e.* pO_2_ > 20%, fully respiratory metabolism). Inlet gas oxygen levels were subsequently stepwise reduced by replacing different air proportions with nitrogen. Thereby either oxygen limited or hypoxic conditions were created in the bioreactor which are characterized by different ethanol and arabinitol production rates [29-31]. Two chemostats were performed for each metabolic steady state and strain.

### Sampling

The different chemostat conditions were maintained for 5 residence times before sampling. As previously reported, this cultivation time is enough to reach a metabolic steady state in *P. pastoris*[32]. For each steady state condition, duplicate samples for intracellular metabolite measurement were taken using the previously described optimized protocol for the direct measurement of *P. pastoris* metabolome [32]. For cellular dry weight, a known volume of cultivation broth was filtered using pre-weighted filters; these were washed with two volumes of distilled water and dried to constant weight at 70°C.

Samples for extracellular metabolite were obtained by rapid sampling of broth with immediate cooling to 0°C and fast filtration, using the cold steel-bead method [37] and analyzed using high-performance liquid chromatography (HPLC) with a Bio-Rad Aminex column at a temperature of 60°C. The mobile phase was 5.0 mM phosphoric acid of which the flow rate was set to 0.6 mL/min.

### Free intracellular amino acid analysis

The intracellular concentrations of Ala, Val, Leu, Ile, Thr, Asn, Asp, Met, Trp, His, Pro, Glu, Gln, Orn, Lys, Tyr and Phe were determined by GC-MS [38]. Briefly, 100 μL of sample were transferred to a glass vial, 30 μL of 100 mg/mL NaCl were added and the mixture was lyophilized. 75 μL acetonitrile and 75 μL of N-methyl-N-(tert- butyldimethylsilyl)trifluoroacetamide (MTBSTFA, Thermo Scientific) were added and the vial was incubated for 1 h at 70°C. Subsequently, the sample was centrifuged (10,000 g, 2 min) and 60 μL of the supernatant were transferred to a GC glass vial with an insert. The sample was then analyzed by GC–MS instrument coupled to a 5975 C MSD single quadrupole mass spectrometer (Agilent, Santa Clara, CA, USA). Glycine levels were also measured in this analysis; however, the obtained results were not included as the values were found to be inconsistent [32]. Quantification was based on isotope dilution mass spectrometry (IDMS) [39]. In total, 17 amino acids were analyzed plus Glycine.

In order to graphically visualize the obtained intracellular quantification together with the central carbon metabolism and amino acid biosynthetic pathways the VANTED software was used [40].

### Antibody fragment quantification

Fab amounts in soluble cell extracts and in culture broths were performed by means of a sandwich ELISA assay as previously described [29].

### Principal component analysis

Reference values for each amino acid pool level were calculated as mean of all measurements independently of the strain or oxygen availability. Afterwards, for each condition, the specific amino acid measurements were divided by the reference value to obtain a free scale value of its variation. These relative fold changes for each condition were illustrated through PCA using the xlstat plug-in software for Excel.

### Determination of energy and degree of reduction for amino acid synthesis

The degree of reduction of each amino acid were calculated using the amino acid molecular formulas as explained elsewhere [41]. Energy costs for the biosynthesis of each amino acid were taken from previously published data for *S. cerevisiae* growing under aerobic conditions [35] as both yeast share equal amino acid biosynthetic pathways [7,8,11].

## Competing interests

The authors declare that they have no competing interests.

## Authors’ contributions

MC performed bioreactor cultivations, experimental data acquisition, data calculation, analysis and interpretation of results and participated in drafting the manuscript. AP and JD performed the GC-MS analysis. JA participated in the overall conceptual and experimental design of this study, interpretation of results and in drafting the manuscript. JH and WG participated in the overall conceptual and experimental design of this study, interpretation of results and in revision of the manuscript. PF participated in the overall conceptual and experimental design of this study, interpretation of results and in drafting the manuscript. All the authors read and approved the final manuscript.

## Supplementary Material

Additional file 1 Comparison of physiological parameters of chemostat cultivations. Click here for file

Additional file 2 Alanine and serine families’ biosynthetic pathways behaviour under different oxygen conditions. Click here for file

Additional file 3 Aspartate and histidine amino acid families’ biosynthetic pathways behavior under different oxygen conditions. Click here for file
