# Supplementary material for: Quantitative metabolomics analysis of amino acid metabolism in recombinant Pichia pastoris under different oxygen availability conditions
Source: Microb Cell Fact. 2012 Jun 15;11:83. doi: 10.1186/1475-2859-11-83 (PMC3538582; doi:10.1186/1475-2859-11-83)
Supplement: Additional file 1 — Comparison of physiological parameters of chemostat cultivations. [file 1475-2859-11-83-S1.doc]

## Additional file 1. Comparison of physiological parameters of chemostat cultivations

Comparison of the macromolecular parameters at steady state of the chemostat cultures performed in this study with those previously reported [30]. q_Gluc_ and q_O2_ are specific utilization rates, and q_X_, q_Ara_, q_EtOH_ and q_CO2_ are specific production rates, where Glc, Ara, EtOH and X stand for glucose, arabinitol, ethanol and biomass, respectively.
