# Supplementary material for: Quantitative metabolomics analysis of amino acid metabolism in recombinant Pichia pastoris under different oxygen availability conditions
Source: Microb Cell Fact. 2012 Jun 15;11:83. doi: 10.1186/1475-2859-11-83 (PMC3538582; doi:10.1186/1475-2859-11-83)
Supplement: Additional file 3 — Aspartate and histidine amino acid families’ biosynthetic pathways behavior under different oxygen conditions. [file 1475-2859-11-83-S3.doc]

**Additional file 3. Aspartate and histidine amino acid families’ biosynthetic pathways behavior under different oxygen conditions.**

The metabolite level bars in the graphs are the average and the standard error from at least 4 measurements. The control and the Fab-expressing strain are represented by black-grey and yellow bars respectively. Oxygenation conditions from normoxic to oxygen-limited conditions are represented as light to dark color scale. The mRNA data are indicated as m plus the specific reaction name. Each graph has its own scale
