# Supplementary material for: Quantitative metabolomics analysis of amino acid metabolism in recombinant Pichia pastoris under different oxygen availability conditions
Source: Microb Cell Fact. 2012 Jun 15;11:83. doi: 10.1186/1475-2859-11-83 (PMC3538582; doi:10.1186/1475-2859-11-83)
Supplement: Supplementary file 3 [file 1475-2859-11-83-S2.doc]

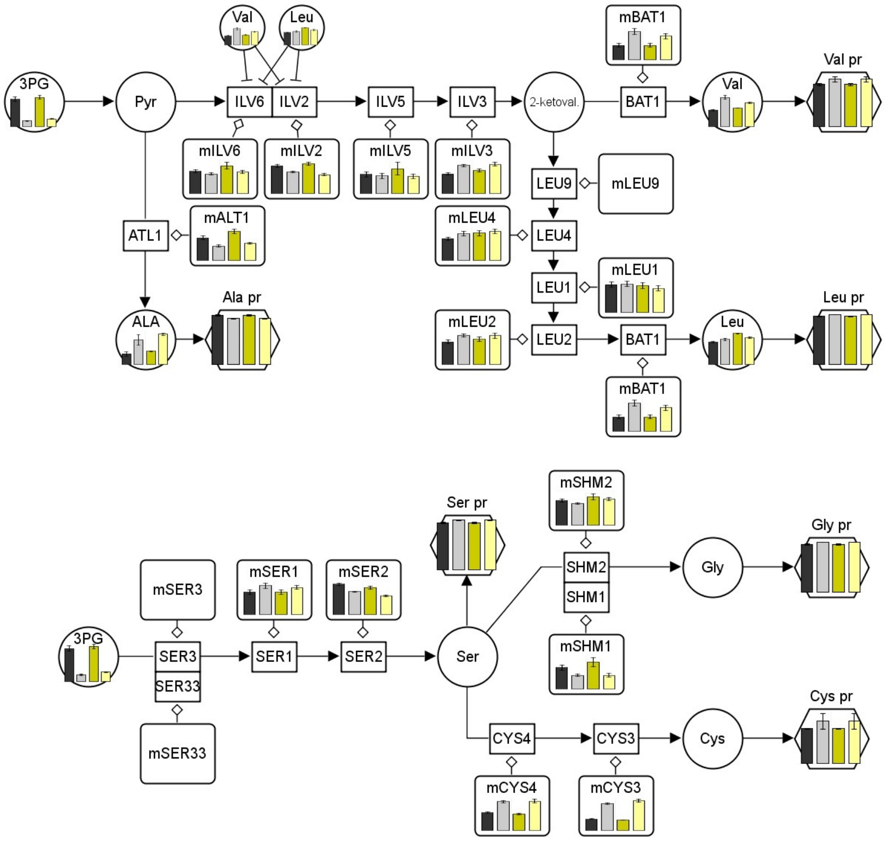

% mol amino acid in the total protein [3]

mRNA levels [1]

Free metabolite pool

Reaction name

Reaction direction

Reaction Inhibition

Reaction Modulation
